# The importance of a developmental perspective in Psychiatry: what do recent genetic-epidemiological findings show?

**DOI:** 10.1038/s41380-020-0648-1

**Published:** 2020-01-20

**Authors:** Anita Thapar, Lucy Riglin

**Affiliations:** 1 Child and Adolescent Psychiatry Section, Division of Psychological Medicine and Clinical Neurosciences, Cardiff University, Cardiff, UK; 2 MRC Centre for Neuropsychiatric Genetics and Genomics, Cardiff University, Cardiff, UK

**Keywords:** Genetics, Psychology

## Abstract

There is growing appreciation that a developmental perspective is helpful in Psychiatry. However, clinical practice and research, especially in an era of very large sample sizes, often ignore the developmental context. In this perspective piece, we discuss why a developmental view is important in Psychiatry and how recent genetic-epidemiological findings further highlight this. DSM-5 childhood neurodevelopmental disorders such as ADHD, typically onset in early childhood but can persist into adult life; the same ADHD genetic loading appears to contribute across the life course. However, recent longitudinal studies have observed that ADHD symptoms may emerge later during adolescence and adult life in some individuals although the etiology of this late-onset group is unclear. The epidemiology and genetics of depression do not appear to be the same in childhood, adolescence, and adult life. Recent genetic findings further highlight this. Autistic type problems and irritability also appear to show developmental variation in their genetic etiology. These findings raise the question of whether social communication and irritability have the same meaning at different ages. Schizophrenia typically onsets after adolescence. However, it is commonly preceded by childhood antecedents that do not resemble schizophrenia itself but do appear to index schizophrenia genetic liability. We conclude that there is a need for clinicians and scientists to adopt a developmental perspective in clinical practice and research by considering age-at-onset and changes over time as well as different developmental periods when interpreting clinical symptoms.

## Introduction

**Fig. 1:**
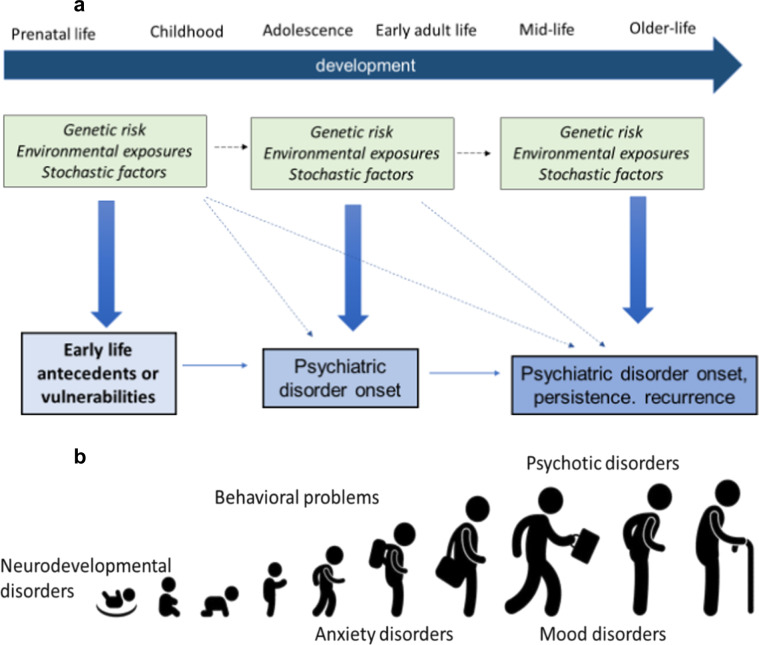
**a** A developmental view of psychiatric disorders. **b** Typical ages-at-onset for different types of psychiatric disorders.

It is well recognized that physical, behavioral, brain, and biological phenotypes are subject to changes across the life span and that such transformation is especially marked during fetal life, childhood, and adolescence. Many of these changes are normative and arise as a result of typical developmental processes (e.g., an increase in height during childhood); others represent departures from a typical developmental trajectory (e.g., a shift from the 50th to 5th centile on a childhood height centile chart). There is growing appreciation from biological, imaging, genetic, and clinical studies that a developmental perspective is important for investigating psychiatric disorders. However, clinical practice and sometimes research, especially in an era when very large sample sizes need to be amassed, often ignore the developmental context. That is because investigating developmental processes and taking a life-course perspective typically require longitudinal investigations that are time consuming and expensive (see Fig. 1a).

The argument that developmental approaches are useful to Psychiatry is not new; this has been argued strongly by Rutter for decades [1]. In this perspective piece, we revisit why a developmental view is important in Psychiatry using recent findings from genetic-epidemiology studies—particularly those using polygenic risk scores (PRS). This article is not intended to be a comprehensive review; rather we have deliberately selected papers, many of which are our own, to illustrate key points for this perspective piece. However, we emphasize that a broad range of psychiatric and applied neuroscience research studies that include investigations of environmental exposures, epidemiological, genetic, transcriptomic and imaging studies [1–4] also illustrate the importance of a developmental perspective but are beyond the scope of this article.

### Timing of onset for different psychiatric disorders

Around 75% of psychiatric disorders onset by childhood, adolescence, or early adult life (mid-20s) [5]. Thus, it can be argued that investigation into risk and protective exposures, risk mechanisms, as well as prevention and early intervention programs needs to start very early in life. Also, the peak age of incidence for many psychiatric disorders, such as depression, coincides with the transition from “childhood/adolescence” to “adult” life. The sharp divide between child/adolescent and adult psychiatry research and clinical services is unhelpful here and can be a barrier to adopting a developmental approach.

Although there is a growing recognition that different neuropsychiatric disorders show strong phenotypic [6] and genetic [7, 8] overlap with each other, they are also distinctive in many respects. An obvious difference is that they display varying times of onset or at least manifest at different times [9, 10] (see Fig. 1b) although what explains this variation is unknown at present. One possibility is that the timing of exposure to a risk factor (e.g., prenatal life vs. adolescence) matters. Also, risk factors vary across the life course. For example, hormonal changes associated with puberty are likely to have greatest impact in adolescence, a time period when the incidence of depression rises. Other stressors also change across development—such as family or school stressors in childhood, employment-related stress in adulthood, and chronic illness in the elderly.

Neurodevelopmental disorders as grouped by DSM-5 [11] include autism, ADHD, learning, communication and motor disorders, and intellectual disability, and are defined as having an onset in the early developmental period, typically in early childhood [12]. They tend to have a steady rather than remitting and relapsing course [10, 11] and their core features show marked maturational changes from childhood to adult life. It is now recognized that these disorders, or at least some symptoms and impairment, persist well into adult life for many.

It has been long recognized that conduct disorder shows distinctive developmental courses. Childhood-onset persistent conduct problems begin early and are strongly associated with neurocognitive deficits, ADHD, a higher genetic loading, and poorer prognosis [13]. In some regards, this group appear to share many similarities with neurodevelopmental disorders: early onset, many but not all show a chronic life-course trajectory, prominent neurocognitive deficits, and a male excess. Another group show an emergence of conduct problem after puberty. Individuals in this group do not show an elevated rate of ADHD or cognitive deficits, which tend to improve in adult life, and the social/peer context is thought to contribute risk in this group [14]. A developmental approach enabled these different groups to be identified, yet clinical research of adolescent conduct disorder would group them as a single entity. Childhood-onset (<10 years) and adolescent-onset behavioral problems however are distinguished as subtypes in DSM-5.

The typical timing for the onset of anxiety disorders depends to a large extent on the type of anxiety problem [15, 16]. For example, separation anxiety and specific phobias typically onset in childhood; social anxiety disorder more commonly arises in childhood and adolescence, and agoraphobia, panic disorder, and generalized anxiety disorder most commonly manifest in later adolescence or early adulthood. Symptoms of some anxiety disorders are developmentally normative at a young age (e.g., separation anxiety in toddlers). As for neurodevelopmental disorders, symptoms of anxiety disorder need to be assessed with a developmental view but there is no clear-cut guidance on how best to do this [16].

Mood disorders and schizophrenia typically rise in incidence from late adolescence onwards and are rare in prepubertal children. However, longitudinal, population-based studies as well as investigation of high-risk offspring of parents with depression, bipolar disorder, or schizophrenia have shown that these disorders are commonly preceded by earlier mental health problems such as anxiety, irritability, or mild hypomania [17, 18]. We will focus on schizophrenia where a developmental perspective has been informative.

### Childhood antecedents of schizophrenia

While the core defining features of schizophrenia typically first onset in late adolescence or adult life (e.g., hallucinations, delusions), it can be preceded by earlier neurocognitive and developmental impairments including language, social, motor, and attentional difficulties in early childhood [10]. These so-called childhood antecedents also have been observed in the high-risk offspring of parents with schizophrenia. It has been unclear however whether these early impairments create risk for later schizophrenia or represent an early manifestation of underlying liability. A recent study examined schizophrenia polygenic risk scores (PRS), a composite measure of the total burden of risk alleles derived from a large patient/control schizophrenia discovery genome-wide association study (GWAS), in a prospective population-based cohort [19]. Schizophrenia PRS were found to be associated with language, social communication, cognitive, and behavioral traits as young as age 4 years. In adolescence, schizophrenia PRS also have been found to be associated with anxiety in the general population [20]. These findings suggest that the antecedents observed in high-risk studies may be the early manifestations of underlying schizophrenia genetic liability, or alternatively these could be causal risk factors.

Studies of prepubertal children carrying highly penetrant rare mutations such as 22q11 microdeletion syndrome also show that even though around 1 in 4 carriers are likely to develop schizophrenia, as children, these individuals show a broad range of psychopathology including ADHD, autism spectrum disorder, anxiety, as well as cognitive impairments [21]. Findings from these studies converge with those from recent gene expression studies. These implicate enrichment of schizophrenia-associated genes identified from GWAS with brain expression profiles manifest during the prenatal period [22]. These findings all suggest that the core defining features of schizophrenia are a late manifestation of an underlying disorder process that starts well before adolescence and adult life.

### Depression: same disorder at different ages?

DSM-5 no longer isolates childhood depression and anxiety diagnostic criteria but rather takes a lifespan approach to classification. There are strong arguments for viewing depression and anxiety as lifespan disorders because child and adolescent forms of disorder appear to show strong links with the same later disorders in adult life. Also, both disorders are familial; the children of parents with anxiety or depression are at elevated risk for these same disorders. These findings suggest that for anxiety and depression, the same underlying liability operates across childhood, adolescence, and adult life.

However, there are some complications to viewing depression and anxiety as a single developmental continuum. First, epidemiological studies consistently show marked differences in the gender ratio for prepubertal depression in childhood and depression in adolescence and adult life. In childhood, males and females are equally affected, whilst after mid-adolescence, females are much more commonly affected [23, 24].

Second, while postpubertal-onset major depressive disorder shows strong continuities with depression in adult life, prepubertal-onset depression does not appear to show this continuity but rather shows links with later antisocial behavior [10, 25]. Third, treatment responses are not identical in children, adolescents, and adults. Tricylic antidepressants while not first line treatment for depression in adults have been found to be an effective treatment; however, one meta-analysis in children found them to be ineffective in this age group. Even serotonin reuptake inhibitors generally have been found to be less effective in young people such that in some countries only fluoxetine is licensed for those under the age of 18 years [26], and they have been reported to be more commonly associated with suicidal thoughts in younger people, [27] so careful monitoring is required. These findings suggest that depression/anxiety are not necessarily the same at different ages. If this is the case, it could have important clinical and research implications.

Finally, turning to etiology, early family and twin studies highlighted that major depressive disorder [28] and depression symptoms [29] also show etiological heterogeneity indexed by age-of-onset or age. More recent molecular genetic studies also are beginning to suggest age-at-onset and age differences in the genetic architecture of depression and we will consider findings from these (see Table 1). The largest GWAS study of depression in patients to date [30] (predominantly adults) found that an early age-of-onset (before 27 years: S Hagenaars and C Lewis, written communication, June 21st 2018) was associated with schizophrenia PRS whereas “later-onset” depression was associated with depression PRS in keeping with a previous study [31].

**Table 1:** Genetic-epidemiology studies investigating depression heterogeneity based on age or age-at-onset.

	Sample		Total *N*	Phenotype	Age-based groups	Early associations	Later associations
Power et al. [31]	PGC MDD	Patient	155,907	Major depression	Early onset (≤27 years) and adult onset (>27 years)	Schizophrenia and bipolar PRS	
Riglin et al. [32]	NCDS	Population	5257	Emotional problems	Childhood (7–16 years) and adulthood (23–42 years)	Schizophrenia PRS	Schizophrenia and depression PRS
Rice et al. [33]	ALSPAC	Population	5416	Clinically significant depressive symptoms	Early-adolescence onset (12 years) and later-adolescence onset (16 years) trajectories	Schizophrenia and ADHD PRS^a^	Depression PRS
Musliner et al. [34]	iPSYCH	Population	34,573	Diagnosis of depression	First diagnosis aged 10–15, 16–20, 21–25, or 26–31 years	Schizophrenia, bipolar, and depression PRS	Schizophrenia and depression PRS

*ALSPAC* Avon Longitudinal Study of Parents and Children, *MDD* major depressive disorder, *NCDS* National Child Development Study, *PGC* Psychiatric Genomics Consortium, *PRS* polygenic risk scores

^a^Multivariable analyses.

Findings from the longitudinal population-based UK 1958 birth cohort, also observed that schizophrenia PRS were found to be associated with a measure of emotional problems (anxiety/depression) across childhood, adolescence, early adult life, and mid-adulthood whereas depression PRS associations were more consistently associated with emotional problems in mid-adulthood [32]. While stronger associations with schizophrenia compared with depression PRS could reflect that the former is a stronger instrument, this does not explain differential findings for depression PRS by age.

A more recent longitudinal UK population-based cohort study focused on an earlier developmental period and investigated depression symptom trajectories from childhood to late adolescence [33]. This study identified a group whose depression symptoms emerged in early adolescence and another group whose symptoms emerged later in adolescence. Both depression groups were associated with depression PRS but interestingly the early emerging depression group additionally was associated with schizophrenia and ADHD PRS. This early-onset depression group also showed elevated levels of childhood ADHD and other neurodevelopmental traits.

Taking together these three studies, we speculate about the possibility that there is a very early-onset form of depression that is characterized by a more prominent neurodevelopmental component. It remains to be seen whether this very early-onset group, like those observed in earlier clinical and epidemiological studies fails to show continuity with typical forms of depression in adult life.

The most recent molecular genetic study of youth depression was based on the Danish iPsych cohort that utilized health record registry data and included those with a clinic recorded diagnosis of depression from age 10 years onwards [34]. These investigators also observed somewhat higher schizophrenia PRS in earlier-onset depression (those aged 10 to 15 years) with MDD PRS showing strongest associations for depression with age-at-onset between 16 and 25 years. They additionally observed that earlier-onset depression was associated with bipolar PRS. This cohort has the advantage of being focused on patients and being very large but of course clinically referred depression is not the same as population-based depression. The authors did not report results for ADHD PRS. It is unknown to what extent these observations reflect pleiotropy (schizophrenia and bipolar PRS increasing risk for depression as well as for schizophrenia and bipolar disorder) or whether some early-onset cases are misclassified as depression and over time will manifest as bipolar disorder or schizophrenia. Regardless, the findings are consistent with multiple other studies in that they vary for depression at different ages.

There are now decades of epidemiological, twin, and new molecular genetic studies as well as pharmacological studies highlighting that a developmental perspective to investigating depression and anxiety will be important for better understanding it and certainly in an era where stratification, precision medicine, and personalized medicine are priorities. That is a big challenge, especially for genomic discovery studies where very large sample sizes are required, individual data and developmental data are typically unavailable.

### ADHD: what happens over time

For DSM-5-defined neurodevelopmental disorders including ADHD, the core symptoms and impairments can show maturational improvements although for many individuals, deficits remain in adult life [10, 11]. For example, ADHD hyperactive-impulsiveness symptom levels decline with age; this age-related decline in inattention problems is less prominent. In acknowledgement of this developmental change, DSM-5 has now reduced the number of ADHD symptoms required in adolescence and adulthood for a diagnosis of ADHD. However, there is little known about typical trajectories of ADHD and other neurodevelopmental disorders in adult life and limited guidance on how to adjust diagnostic assessments according to the developmental context in either clinical practice or research. This is important given that longitudinal studies of ADHD [35], autism [36], language [37], reading [38], and spelling problems [39] show that a substantial proportion of affected individuals continue to meet full diagnostic criteria in adult life or at least display some symptoms and manifest impairment in adult life. These findings are a reminder that many individuals with childhood neurodevelopmental disorders do not grow out of them as was a common belief previously nor do they represent a maturational lag [40] whereby affected children eventually “catch up.” However, alterations in the developmental context (for example, not being required to sit in school exams) may mean that the nature and level of difficulty and impairment changes. Nevertheless, there is substantial variability in individual developmental trajectories and the reasons behind this are largely unknown yet may provide some clues for optimizing outcomes. Here we take the example of ADHD.

Family and twin studies all suggest that stronger familial and genetic loading for ADHD are associated with ADHD continuity into adolescence and adult life [41–43]. A recent, longitudinal, population-based study examined repeated measures of ADHD symptom scores from ages 4 to 17 years and association with ADHD PRS [44]. Overall ADHD symptom scores declined for the majority; but two groups differentiated those with a persistently high level of ADHD symptoms (through to adolescence) and those whose symptoms declined or remitted by adolescence (childhood-limited). ADHD PRS distinguished the high persistent group from the childhood-limited group (and from the other groups). Interestingly although there is such strong genetic overlap between different neuropsychiatric disorders, schizophrenia, bipolar and major depression PRS were not associated with the ADHD developmental trajectories.

PRS are unavailable in clinics and in any case are only weakly predictive. However, an index of neurodevelopmental and behavioral multimorbidity—lower IQ, autistic problems, language problems, and conduct problems—also were observed to be associated with ADHD PRS burden and independently predicted a persistent trajectory. These findings overall suggest that the genetic contribution to childhood ADHD also contribute to its persistence in adult life. More recently, independent genome-wide association studies of adult ADHD and child ADHD also showed strong genetic correlation between adult ADHD and child ADHD [45]; again suggesting that while ADHD symptoms might show developmental change, the genetic correlates do not appear to be very different.

Interestingly, several recent longitudinal studies adopted a developmental life-course perspective to assessing ADHD and identified an apparent “late-onset” form of ADHD where ADHD symptoms were not reported in childhood but emerged newly in adolescence or adult life [46]. These individuals would not meet current diagnostic criteria for ADHD if their age-at-onset was known and the discovery of this group challenges the conceptualization of ADHD as a neurodevelopmental disorder. Three studies investigating “late-onset” ADHD have not observed associations with ADHD PRS, although these have been limited in power due to sample size [44, 47, 48]. Thus, while childhood and persistent ADHD appear to be genetically similar, this may not be the case for “late-onset” ADHD. Further work is needed on this late-onset group.

### Autistic symptoms in childhood and adolescence

It has been known for a long time from family and twin studies that autism is familial, highly heritable, and genetically associated with a broader spectrum of social and communication difficulties [49, 50]. More recent molecular genetic studies also have highlighted that clinically diagnosed autism shows genetic overlap with broadly defined social communication traits in the general population [51]. Autism, like other child neurodevelopmental disorders, is not restricted to childhood and many affected individuals continue with persistent problems into adult life. However, emerging findings from one recent longitudinal cohort study suggest that social communication traits not only show change in their phenotypic manifestation over time but also in their genetic architecture [52].

This UK population-based cohort used psychiatric GWAS findings to examine the latent genetic architecture of social communication traits from ages 8 to 17 years. The authors found that while there was some genetic stability across ages, most of the genetic contribution at age 17 years came from a novel genetic factor at age 11 years, highlighting novel adolescent processes [52]. The same research group also found that while autism PRS showed association with social communication traits in the general population in childhood, this association declined with age [53].

Interestingly, social communication problems were also found to be associated with schizophrenia PRS; however, this association was strongest in late adolescence. The findings raise the possibility that the meaning of social communication problems may be different across ages but further work on this is needed.

### Can a developmental perspective help classify or subtype psychopathology? The example of irritability

In this next section we consider the clinical phenomenon of irritability: specifically, whether it is best conceptualized as a mood, behavioral, or ADHD-like neurodevelopmental problem. This knowledge could help shape clinical management in the future. Severe irritability, commonly defined as increased anger relative to peers, is a common reason for referral to clinical services and is a growing management problem to clinicians [54]. It is transdiagnostic [55] and has been a topic of some controversy because of uncertainty as to how it should be conceptualized in childhood. Irritability is a feature of the behavioral problem, oppositional defiant disorder (ODD) and factor analyses have shown that ODD separates into distinctive irritable, headstrong, and hurtful dimensions [56]. The most recent ICD-11 includes irritability as a specifier of ODD. However, in DSM-5, chronic, severe childhood-onset irritability is classified as disruptive mood dysregulation disorder and under mood disorders. The rationale for this comes from epidemiological studies that have observed prospective association between irritability and later depression and a twin study that showed high genetic overlap between irritability and depression [57].

However, irritability and a closely related construct, emotional lability, are also strongly associated with ADHD. Indeed, irritability was once considered a core feature of early conceptualizations of ADHD [58]. The view that irritability is closely related to ADHD is strengthened by findings from a twin study that showed genetic overlap between emotional lability and ADHD and cohort and patient studies that have found that irritability is associated with ADHD PRS [59, 60].

Thus, it is difficult to resolve how best to conceptualize irritability. A developmental perspective could help here.

In a recent longitudinal cohort study conducted by us, irritability was assessed repeatedly over childhood and adolescence. Irritability symptom levels were high for males in childhood (higher than females) but then declined in adolescence. The pattern was different for females in whom irritability levels increased in adolescence (higher than males) [60]. In further analyses, using growth mixture modeling, the longitudinal data was used to class different irritability groups that differed in their ages-of-onset [61]. One group was characterized by a childhood onset, elevated level of irritability that was more common in males. This group was associated with higher levels of both ADHD and depression and also associated with ADHD PRS (even when those with a diagnosis of ADHD were excluded). Another group showed irritability onset in adolescence and a female preponderance. This group was associated with a diagnosis of depression and depression (as well as ADHD) PRS.

These findings require replication but we hypothesize that irritability might be both ADHD-like (or ODD-like) and depression-like but that developmental context could be important in differentiating irritability types. That could have important future implications for treatment and classification systems.

### Considerations for the future: challenges and potential ways forward

The idea of a developmental perspective in Psychiatry being important is not new. However, in this perspective article, we focused on recent genetic-epidemiological findings, particularly GWAS and PRS findings. While, this research has limitations, when findings are taken together with those from other research designs, they once again highlight the importance of a developmental perspective. Differential PRS associations for what appears to be the same phenotype, depending on age or age-at-onset, suggest possible etiological differences across development. Such findings may help refine phenotype definitions and inform potential stratification. The findings are also relevant to clinicians who will need to consider age-at-onset and developmental changes over time as well as different developmental periods when interpreting a given clinical presentation. Traditionally, there has been a tendency to ignore developmental issues in clinical practice and psychiatric research and this might in part be due to a variety of different challenges. One challenge lies in the measurement of psychiatric disorder. While categories can be useful for clinical decision making, psychiatric disorders appear to lie at the extreme of continuously distributed dimensions [62]. Dimensional approaches including R-Doc do have many advantages. However, these assume that any given dimension, for example negative valence, has the same meaning in early childhood as early adult or mid-life for example.

Future important questions include do the same symptom, construct, dimension mean the same thing at different ages? This could be tested in a variety of ways, for example by assessing etiology, neural correlates, future trajectories, and outcomes.

A second challenge is the divide between child/adolescent and adult research and services. For example, different measurement approaches are used in childhood/adolescence and adult life including different types of measure and informant (e.g., parent, teacher vs. self). Longitudinal studies that attempt to utilize the same sorts of measures across ages especially across the adolescent/adult divide could help with investigating important questions about the natural history of psychopathology, continuities, and discontinuities across time, when trajectories are altered and how these are affected by environmental exposures. Transition clinical services that bridge adolescence and young adult life could also help address the divide.

Issues that require further investigation include testing whether age-at-onset helps distinguish clinically useful subgroups in terms of selecting treatments and predicting prognosis. Can we consider some mental disorders as deviant developmental trajectories and if so, what is the natural history of these trajectories and what modifiable factors alter them and when? Are there differences across different countries that could provide clues about causal risks and prevention or intervention targets?

A third challenge lies in obtaining reliable data across the life span. Unfortunately, retrospective reports for dating onset of disorder and for recalling early symptoms [63] and environmental exposures [64] are unreliable. Ideally developmental studies require longitudinal studies that start in very early life and continue through to late adulthood.

Given growing evidence of the importance of early brain development in relation to psychiatric disorder, prospective pregnancy-birth cohorts are especially attractive. Such studies have shown enormous value because they start before the onset of disorder, focus across different diagnoses, typically take a dimensional as well as a categorical approach to measurement [65] and include multiple risk factors measured across the life course. However, prospective cohort studies suffer from nonrandom attrition and are less suited to investigating rare exposures (e.g., very preterm birth) and less common outcomes (e.g., schizophrenia, bipolar disorder). However, prospective high-risk studies (e.g., offspring of parents with mental illness; enriched for a known risk factor such as rare mutation or preterm birth) as well as nationwide prospective clinical registry data can be useful additions here.

## Conclusions

We emphasize in this article that a developmental perspective is important for all clinicians and researchers spanning from neuroscientists, geneticists, and epidemiologists to those conducting treatment trials and that this approach is currently timely. It does bring challenges for our measurement of psychopathology, classification and research framework, as well as research design and services but there are some solutions. We urge for a greater focus on developmentally orientated research, for clinicians to adopt a developmental perspective in clinical practice and for scientists to bring a developmental perspective to a broad range of science.
